# Children's syntactic representation of the transitive constructions in Mandarin Chinese

**DOI:** 10.1371/journal.pone.0206788

**Published:** 2018-11-07

**Authors:** Dong-Bo Hsu

**Affiliations:** Department of Chinese as a Second Language, National Taiwan Normal University, Taipei City, Taiwan, R.O.C.; Pennsylvania College of Health Sciences, UNITED KINGDOM

## Abstract

Two studies are presented that investigate the effect of linguistic cues on Mandarin speakers’ comprehension of transitive constructions. Study 1 investigated Mandarin-speaking 2- to 4-year-olds’ and adults’ comprehension of the SVO (Subject-verb-object) construction, *ba*-construction (SbaOV), and subjectless *ba*-construction ((S)baOV) with novel verbs using the forced choice pointing paradigm (FCPP). Study 2 investigated another group of participants with similar ages’ comprehension of the SVO construction, the *ba*-construction, the long and short passive constructions with novel verbs and FCPP. Although these constructions have differing cue strengths, participants in the same age groups comprehended these construction types equally well. The results suggest that children as young as two attended to the case markers of *ba* and *bei*, allowing them to employ abstract syntactic representations in comprehending Mandarin transitive constructions. The findings demonstrate that children are sensitive early on to the structural information encoded in the constructions.

## Introduction

Over the past decade, one of the most hotly debated topics in developmental psycholinguistics has centered on whether young children have adult-like syntactic representations that they employ in language processing and that are independent of the input to which they are exposed [1,2]. The current study aimed to investigate how Mandarin-speaking children’s and adults’ comprehension of transitive constructions is influenced by syntactic representations and input characteristics, given that Mandarin allows for massive argument ellipsis and various word orders [3], whereas arguments are obligatory, and SVO is the predominant word order in English transitive constructions [4].

In discussing the issue of whether young children have adult-like syntactic competence, developmental psycholinguists who are proponents of the structure-mapping theory [5–12] and those who are proponents of the usage-based theory [1, 2, 13–19] have held different theoretical points of view on how and when young children acquire their syntactic knowledge. Thus, these two theories have generated different hypotheses concerning young children’s demonstration of syntactic competence.

Fisher and colleagues argued that young children can use structural knowledge, such as the number of nouns and word order, to construct a partial syntactic representation that constrains verb interpretation, which then promotes subsequent verb learning. This process is called ‘syntactic bootstrapping’. The establishment of this partial syntactic representation is derived from a structure-mapping framework, which is based on the following three assumptions. First, young children can use the number of nouns in a heard sentence to estimate probabilistically the arguments/participants that the verb requires in the conceptual structure via a structural analogy between syntactic structure and semantic structure. Children begin with an unlearned, biased one-to-one mapping between nouns and participant roles in an event. Second, children are biased to abstract away the linguistic details to more general conceptions such as agent and patient, as opposed to word-specific representations [19]. For example, although *the dog* in “The dog pushed the cat” and “The dog pulled the cat” have many different features, e.g., pusher vs. puller, children tend to represent these two characters more abstractly, i.e., as agents. Third, through an unlearned bias linking the number of nouns and a biased usage of word order knowledge that treats the first noun phrase (NP) as the agent and the second NP as the patient, children treat the heard information using an abstract mental vocabulary and establish a partial syntactic representation <NP1-Agent, Verb-transitive/causal, NP2-Patient> to guide their interpretation of verbs. Therefore, this theory predicts that the generalization of verbs should be rapid and should occur early in language acquisition, without the accumulation of exemplars to form the abstraction of agent, patient, and so on. Evidence that children as young as 19–29 months old can successfully map the relationship between the agent and the first NP and the patient and the second NP using novel verbs in the intermodal preferential looking paradigm (IPLP) suggests that young children are equipped with abstract syntactic and semantic structures and innate one-to-one mapping between the two. This mapping enables them to use abstract knowledge to constrain the meaning of novel verbs [9, 12, 20, 21]. Hence, toddlers are able to demonstrate their productive knowledge at an early age. However, toddlers might not be able to demonstrate syntactic competence in experimental tasks if: (a) the task requires unavailable executive commands; and (b) cognitive processing ability and memory are limited [6].

Conversely, Abbot-Smith, Lieven, and Tomasello [13] denied the possibility of the rapid formation of abstract agent and patient roles and of the innate one-to-one mapping of thematic roles to corresponding NPs. They argued that, in young children, representations of productive knowledge are graded in strength. The strength of the representations depends upon the number of exemplars in the children’s linguistic input. The ease of acquisition of the form-meaning mapping system depends upon the relative learnability of the subject and the object markers that signal the agent and patient in a particular language. Only strong representations allow for a clear signal to other parts of the cognitive system and thus provide clear evidence of productive knowledge through form-meaning mapping. In contrast, weak representations only allow for productive knowledge to be demonstrated for dependent measures that do not tax executive mechanisms and memory systems, such as the IPLP. Hence, these authors made two major claims concerning young children’s demonstration of productive knowledge: (a) poor performance on demanding tasks occurs because of weak representations, as a result of insufficient/impoverished input; and (b) the relative learnability of formal cues in the input influences the development of subject-agent to object-patient relationships. Evidence that young children acquire frequent verbs earlier than infrequent verbs [1,2,22] and that young children demonstrate abstraction better in act-out studies than in elicitation production studies and better in IPLP than in act-out measures [17] lends support to the usage-based theory. In addition, that young children produce constructions that are more prototypical, i.e., supported by redundant cues, such as animacy and word order, working in coalition to identify the agent (e.g., “The dog bit the bone”), than constructions that are less prototypical—i.e., only one cue, namely, word order, is used to identify the subject (e.g., “The dog bit the fish” [1])—lends further support to the usage-based models, such as graded representations theory of young children’s language acquisition [23].

To resolve the debate between the structure-mapping theory and the graded representations theory and to observe how these two accounts can be applied to the acquisition of syntax among Mandarin-speaking children, we investigated five types of Mandarin transitive constructions. They were SVO construction, *ba*-construction, subjectless *ba*-construction, long passive construction, and short passive construction. The SVO construction *qie chidiaole yu* (“The penguin eats the fish up”), which consists of a subject, a verb compound and an object, is parallel to the English SVO constructions. This construction has an agent-patient order.

The *ba*-construction *qie ba yu chidiaole* (“The penguin eats the fish up”) consists of a subject, an object case marker *ba*, an object, and a verb compound, and in contrast to the typical Mandarin word order [24], the Mandarin *ba*-construction has an SOV word order. It also has an agent-patient order. Mandarin is a language with massive noun ellipsis, particularly for the subject [3,16]. Therefore, a *ba*-construction without the subject *ba yu chidiaole* (“Something/someone eats the fish up”), which consists of the *ba* marker, the object, and the verb compound, still allows a Mandarin speaker to distinguish who the agent is and who the patient is. That is, the *ba*-marked NP is explicitly identified as the patient.

Mandarin has two types of *bei*-passive constructions [25], exactly like English has be-passive and get-passive. One is called a long-passive construction *yu bei qie chidiao le* (“The fish was eaten up by the penguin”), which has a patient NP *yu* ‘fish’ preceding the *bei*-marker and an agent NP *qie* ‘penguin’ following this marker, followed by the compound verb *chidiao* ‘eat up’ plus the aspectual marker -*le*. It has a patient-agent order. The other is called a short passive construction *yu bei chidiao le* (“The fish was eaten up”), in which the agent NP *qie* ‘penguin’ in its long passive counterpart is omitted, or it allows for an unmentioned agent, exactly like the by-phrase in the English passive can be unmentioned. It has a patient first order marked by *bei*.

Because Mandarin is an isolating language with a morphological system that is impoverished [3], whether Mandarin has a case system like German or Greek remains subject to debate. Nevertheless, Chinese linguists [25] have argued that Mandarin has a case system and that *ba* as a patient-(object) case marker and *bei* as an agent case marker are exceptions to this impoverished system [3]. Huang, Zheng, Meng, and Snedeker [26] confirmed that the markers *ba* and *bei* are used to exclusively identify the patient and agent, respectively, according to their analysis of sentences featuring *ba*- and *bei*-constructions from the Lancaster Corpus of Mandarin Chinese [4].

To establish the link between input and young children’s comprehension performance and to quantify the relative learnability of formal cues, namely, cue strengths, we followed Kemp and MacWhinney’s [27] formula for calculating cue availability, cue reliability, and cue validity for the cues of word order and case marking with *ba* and *bei*, which can be used to identify the agent and patient. Cue availability refers to the number of cues that are present divided by the total number of the transitive utterances coded above. Cue reliability is defined in terms of the proportion of the presence/availability of a cue that correctly indicates a particular function, namely the agent or patient. Cue validity refers to the product of cue availability and cue reliability. Therefore, the calculations of the cue strengths for these Mandarin transitive constructions are specified.

It has long been noted from corpus studies that SVO constructions are predominant in Mandarin Chinese, and the *ba*-construction constitutes one-tenth of the SVO constructions in number [24], and Huang et al. reported that *bei*-constructions are fewer than their *ba*-counterparts. Huang et al. [25] analyzed 3396 sentences featuring *ba* and *bei* from a search of 20376 sentences from the Lancaster Corpus of Mandarin Chinese, and they reported that the cue availability for the *ba*-construction was 836/20376 = .041, for the subjectless *ba*-construction was 1147/20376 = .056, for the long *bei*-construction was 505/20376 = .025, and for the short *bei*-construction was 747/20376 = .037. Since these two markers exclusively mark patient and agent in Mandarin, their reliability is 1. As a result, their cue validities equal their cue availabilities. In contrast, Chan et al. [16] reported that the cue validity for the SVO construction falls between .30 and .44 depending on how the word order cue is used for the calculation. The cue validities of these five types of Mandarin Chinese transitive constructions are summarized in Table 1.

**Table 1:** Cue validity for transitive constructions in Mandarin Chinese.

	Constructions				
	SVO	SbaOV	baOV	SbeiOV	SbeiV
Validity	.30-.44	.041	.056	.025	.037

This paper employs two sets of constructions to tease apart the predictions of the structure-mapping account and graded representations to account for and to avoid the effects of fatigue that can affect younger groups when five types of transitive constructions are simultaneously administered to them. Study 1 investigated the Mandarin comprehenders’ performance using a set of sentences that consisted of the SVO construction, the *ba*-construction, and the subjectless *ba*-construction. Study 2 investigated another group of Mandarin comprehenders’ understanding of the other set of sentences, which are composed of the SVO construction, the *ba*-construction, the long-passive construction, and the short-passive construction.

The use of the SVO construction, *ba*-construction, and subjectless *ba*-construction in Study 1 to investigate young children’s comprehension helped resolve the debate between these two theoretical perspectives regarding their employment of syntactic abstraction in comprehending transitive constructions. The SVO construction and the *ba*-construction are both compatible with the <1^st^ NP-Agent, 2^nd^ NP-Patient> representation, but they differ in terms of construction frequencies. The subjectless *ba*-construction has a reliable case marker *ba* to denote the patient, although its construction frequency is as low as its counterpart that has a subject. If the syntactic representation and structural information as a reliable case marker facilitate young children’s comprehension regardless of input frequencies, then children should comprehend these three constructions equally well. In contrast, if the input frequencies determine young children’s comprehension, then they should comprehend these three constructions differently with much better comprehension of the SVO construction than the *ba*-construction and the subjectless *ba*-construction.

Study 2 was designed to test the other set of constructions: the SVO construction, *ba*-construction, long-passive construction and short-passive construction by investigating how children within the same age range comprehend constructions with frequencies that are low, such as the *ba*-construction and the short- and long-passive constructions in Mandarin, but that have a different arrangement of the representations of the thematic roles, i.e., one consists of <NP1-agent, NP2-patient> whereas the other consists of <NP1-patient, NP2-agent>.

Study 2 replicates the results of the SVO construction and the *ba*-construction obtained in Study 1 by including these two constructions. Moreover, Study 2 compares the *ba*-, long-passive and short-passive constructions in Mandarin to allow us to understand when and how Mandarin-speaking preschoolers comprehend these low-frequency constructions [4,24] and to tease apart the aforementioned accounts. These three types of constructions occur with very low frequency in Mandarin. Therefore, the graded representation accounts might predict that Mandarin-speaking children are unable to understand these two constructions before the age of 3.5 years old [18,19], whereas the structure-mapping account would predict that Mandarin-speaking children comprehend the *ba*-construction better than the *bei-*construction because the former is consistent with the <NP1-agent, NP2-patient> tendency, whereas *bei*-constructions are not.

Conversely, based on the input statistics discussed earlier and the cue validities summarized in Table 1, several different predictions can be made based on the different foci of the input characteristics. If cue availability is the most important characteristic that affects young children’s performance, but cue reliability plays a more critical role for older children and adults [28], then two predictions can be generated for young children and for older children and adults, respectively. Mandarin-speaking children should perform better on the transitive constructions with higher cue availability; thus, their performance on these transitive constructions should be SVO > *ba*-construction = subjectless *ba*-construction > = short-passive construction = long-passive construction, whereas older children’s and adults’ performance should be (S)*ba*OV = S*ba*OV = long-passive construction = short-passive construction > SVO construction. If cue validity plays a critical role in the comprehender’s demonstration of syntactic abstraction [3,16], then different cue validities for these five transitive constructions should affect the listener’s/comprehender’s performance. The prediction is thus SVO > *ba*-construction = subjectless *ba*-construction > = short-passive construction > = long-passive construction. Language users, particularly young children, might perform best on prototypical constructions, i.e., constructions in which cues work together to identify agent-patient relationships, and perform worse when cues are in conflict [1,3,16,23]. If so, then the language user’s performance should be *ba*-construction > SVO = subjectless *ba*-construction = long passive construction = short passive construction because, although the short and long-passive constructions are constructions in cue conflict, the short- and long-passive constructions still have a salient *bei*-marker to resolve this conflict. Nevertheless, if young children’s syntactic abstraction is influenced by their representation of <1^st^ NP-Agent, 2^nd^ NP-Patient>, then they would perform equally well on the SVO construction and the S*ba*OV construction and perform worse on the long passive construction and short passive construction [21]. Concerning the *ba*OV construction, Naigles [29] argued that toddlers and young children are good at form tracking when acquiring syntax. If this argument is correct, once these young children use abstract representations to comprehend the S*ba*OV construction, they can easily detect the correlates between *ba* and the patient role assigned to the NP that follows *ba* plus this marking following the agent-patient order, and thus they can rapidly employ this representation to comprehend the subjectless *ba*-NP. As a result, the children can perform equally well on the *ba*-construction and the subjectless *ba*-construction. To test these predictions, Study 1 tests Mandarin-speaking children aged from 2 to 4 years old and adults for their comprehension of the SVO, *ba*-, and subjectless *ba*-constructions when animacy is neutralized. Study 2 tests these children’s and adults’ comprehension of the SVO construction, the *ba*-construction, long passive construction and short passive construction when animacy is also neutralized.

The 2 studies used the forced-choice pointing paradigm (FCPP), which is argued to be a better methodology to investigate syntactic abstraction in sentence comprehension when both toddlers and adults are studied [30] and when novel verbs are used to investigate these constructions. When a familiar verb, such as *push*, is used in an SVO construction, young children might be able to use their established, lexically specific knowledge to interpret the sentence to complete the event of pushing; a *pushee* and *pusher* are required, and the *pusher* is always mentioned earlier in the event. In other words, we cannot know whether young children can extend this knowledge to verbs other than *push*. As a result, using novel verbs provides us with compelling evidence about toddlers’ level of syntactic abstraction in a verb-general manner.

In addition to allowing us to tease apart the predictions illustrated above, using these Mandarin transitive constructions can also serve the following purposes. First, Mandarin is a well-known argument-ellipsis language [3,16,31]. However, it remains unclear how massive argument ellipsis affects young Mandarin-speaking children’s comprehension of sentences, such as the SVO construction, the SbaOV construction, and the long passive construction, which have full argument realizations, and the subjectless *ba*-construction and the short passive construction, which can be comprehended when an argument is elided. Second, this study attempts to direct the focus of earlier studies of Mandarin toward an investigation of how young children and adults process structural information, i.e., when the animacy information is neutralized in sentence comprehension [1], because earlier studies have often drawn attention to how animacy affects sentence comprehension, most likely because Mandarin is considered an animacy-dominated language [3,16]. Third, recent studies [2,3,7,16,21,30] have reported that young children are able to demonstrate abstract competence in the SVO construction at as young as 21 months old. However, it remains unclear whether toddlers demonstrate abstract syntactic competence in other types of constructions. To prove the existence of this behavior, we must provide evidence that young children not only use this abstract knowledge to interpret the SVO construction, but they can also extend this knowledge to other types of transitive constructions [30,32], such as the *ba*-construction, the subjectless *ba*-construction, and long and short passive constructions in Mandarin. It is expected that these studies could illuminate the processes that occur in Mandarin speakers when they use these two structural cues to comprehend Mandarin syntactic transitive constructions, and they could provide evidence to illuminate the issue of whether young Mandarin children have abstract syntactic competence.

## Experiment 1

The issues of when and how young Mandarin-speaking children can employ a verb-general syntactic representation to interpret transitive constructions like adults do and whether they can extend this ability to constructions other than SVO are matters of intense debate. Part of this debate includes how cue strength in young children’s input affects their syntactic representation of abstraction, as outlined above [32]. Studies have reported that Mandarin-speaking two-year-olds demonstrate adult-like syntactic competence when familiar verbs are used, as shown in both corpora [33] and experimental studies [21,34,35]. However, whether Mandarin-speaking two-year-olds can demonstrate abstract syntactic competence when novel verbs are used is unclear. Chan et al. [16] investigated how Cantonese-speaking two-and-a-half-year-olds (broadly defined as Chinese) employed cues of word order and animacy to comprehend SVO constructions with novel verbs, using act-out measures. The authors found that these children demonstrated abstract syntactic competence in the cue-in-coalition construction (i.e., both word order and animacy that support the actor/agent, as in “The chicken tams the present”). However, they failed to do so in one-cue construction (i.e., word-order-only condition, as in “The cow meeks the giraffe”) or in cue-in-conflict construction (i.e., in which word order indicates the first NP as the agent, but animacy indicates the second NP as the agent, as in “The present tams the chicken”). Their findings suggested that Cantonese/Mandarin-speaking two-and-a-half-year-olds do not have an adult-like syntactic competence and that they learn to achieve adult-like competence through the accumulation of exemplars in input after the age of 3.6 years old, as the usage-based theory suggests [18]. The authors also found that Cantonese-speaking 3- and 4-year-olds used overgeneralized word order cues to interpret the IVA (Inanimate-Verb-Animate) construction, in which the inanimate NP precedes the animate NP, as in “The present tams the chicken”, and this arrangement leads the word order cue and animacy cue into conflict, an area in which Cantonese-speaking children perform differently from adults [3]. It is also unclear whether Mandarin-speaking preschoolers would use strategies of comprehension that differ from adults in terms of the *ba*- and subjectless *ba*-constructions when the animacy cues are neutralized.

To investigate the issues of whether Mandarin-speaking two-year-olds have abstract competence, we must consider the experimental tasks, the nature of the syntactic constructions, and the adults’ performance. First, Dittmar et al. [1] reported that when investigating two-year-olds’ demonstration of syntactic competence, they found that act-out measures remained too demanding. They reported that German-speaking two-year-olds who could not demonstrate syntactic competence in act-out measures could do so when FCPP was used. Second, Naigles [29] argued that young children are good at detecting form and generalizing structural patterns, but they have difficulty integrating semantic information into their existing linguistic representations. Because animacy is a semantic cue that young children require to integrate relevant semantic information when they are demonstrating their syntactic competence, we must use syntactic/structural cues, such as word order and case marking, to investigate young children’s syntactic competence [1]. Third, in asking whether young children, such as two-year-olds, have adult-like syntactic competence, another means of exploring this issue is to investigate directly the relevant demonstration throughout children’s lifespans. If they are processing grammatical information, i.e., using cues to interpret grammatical function as adults do, then we might also obtain evidence to show that young children can demonstrate adult-level syntactic competence, even in a less demanding task such as FCPP, which has been hypothesized to be suitable only for weak and abstract representations. Fourth, Li et al. [3] reported that Mandarin-speaking adults are not good at using *ba* as an objective case marker with familiar verbs due to its low cue availability and cue validity in input. It is unclear how Mandarin-speaking adults will respond to the *ba*-construction and subjectless *ba*-construction with novel verbs and whether young children will respond to them similarly.

This study tests the predictions derived in the introduction by investigating how Mandarin speakers aged two to four years old and adults comprehend the aforementioned three Mandarin transitive constructions when animacy cues are neutralized using FCPP.

## Methods

### Participants

Forty-two 2-year-olds (range = 2;0–2;10, mean age = 28.21 months, SD = 2.32, 21 boys), 42 3-year-olds (range = 2;11–3;6, mean age = 40.76 months, SD = 2.86, 19 boys), 42 4-year-olds (range = 3;7–4;10, mean age = 54.93 months, SD = 2.60, 17 boys), and 42 adults (range = 19–36, mean age = 25.88, SD = 3.74, 17 men) participated in a language comprehension task using FCPP. All of the participants were speakers who were dominant in Mandarin Chinese, with less than 20% exposure to a language other than Chinese dialects, and they had no language difficulties on the basis of questionnaires for the children’s biodata completed by their caregivers or parents. The child participants were recruited in nurseries, kindergarten, and the children’s own homes. The adult participants were recruited at two national universities in Taipei, Taiwan. All of the participants were tested in sound-dampened rooms, either in the labs at the two national universities or in their nurseries, kindergartens, or own homes. After the experiment, the child participants were given a pamphlet of cartoon stickers, and the adult participants were paid NT$100. To be included in the final sample, all of the participants had to meet the following criteria: (a) 75% (> = 6 animals) in the animal identification phase; (b) 2 out of 3 intransitive novel verb trials; (c) 2 out of 3 familiar verb trials; and (d) at least one test trial. Twelve 2-year-olds, 9 3-year-olds, 3 4-year-olds, and 1 adult were excluded because they did not meet the criteria above. Another 3 2-year-olds were excluded due to fussiness.

This study involving human participants has been approved by the author’s Institutional Review Board (IRB) in Taiwan (Research Ethics Committee, National Taiwan University). The approval numbers are NTU-REC No.: 201312ES034 and NTU-REC No.: 201506ES028. Informed written consent has been obtained from the participants for the adult participants and from the child participants’ parents.

### Materials, design, counterbalancing, and procedures

Hsu [36] used the following method which was identical to the one used here to probe how Mandarin-speaking adults and L1 English Mandarin L2ers’ and L1 Japanese Mandarin L2ers’ develop their syntactic representation when animacy cues were neutralized and semantic information was impoverished. All but some modifications that were tailored to young children’s development were used in the following two studies.

All of the verbs referred to prototypical causative-transitive actions with clear end points. These actions involved a volitional agent exerting some effect on a patient, either through use of a tool or through direct contact. All of the actions were reversible, thus permitting for a manipulation such that two animal characters in the two synchronized animations could be flipped with identical actions. The three novel verbs *fo*, *lei*, and *duain*, all of which have a first tone, were used to describe three novel transitive actions with a clear end state that were performed by two animal characters. *Fo* was used to refer to an animal placing another animal on a crescent-like apparatus and throwing it so that it fell to the ground. *Lei* referred to an animal pushing another animal standing on a turning ball and allowing it to fall. *Duain* referred to an animal pulling down by its leg another animal standing on a rock and allowing it to fall to the ground.

The agents and patients of particular events were pairs of animals familiar to all of the participants, including the two-year-olds. These animals included a rabbit, hippo, dog, lion, cat, tiger, bear, and monkey. No participants showed any difficulty in identifying these animals. These animal characters were used to generate cartoon animations, which were created in Flash Player and exported as movies to Dreamweaver. The movies consisted of two synchronized side-by-side animations and were presented twice on a laptop computer. The events in these two synchronized animations were identical except for when the animal characters were flipped.

The participants heard the same test sentences with the construction types counterbalanced; these sentences were recorded by a male native Mandarin speaker and were exported into the Dreamweaver movies. Therefore, all of the participants heard three novel verbs, each paired with a type of transitive construction, demonstrated by two pairs of animal characters. That is, there were six trials for these three novel verbs and three types of transitive constructions. The participants in the test trial heard each of the following three types of construction with the novel verbs, e.g., *xiaogou fole xiaomao*, *xiaogou ba xiaomao fole*, ‘A dog foed a cat’, and *ba xiaomao fole*, “Something foed a cat”. In each test trial, the novel verb was paired with two sets of animal characters. For each set of animals, the sentence that described the event with this set of animals was presented twice in each of the three different types of Mandarin transitive constructions. For example, the participants heard the novel verbs presented in the SVO construction (e.g., *xiaogou fole xiaomao*, “A dog foed a cat”), and the sentence was repeated. After the presentation of each sentence, a prompt such as *Zhizhe xiaogou fole xiaomao de difang* (“Point at the location where a dog foed a cat”) was provided. After the presentation of the first set of animations, the second set of animations was paired with the same type of construction, and the test sentence was repeated.

The FCPP is a comprehension task that utilizes pointing. Participants were thus required to point to one of the synchronized animations presented on the screen of a laptop computer. Both animations involved animals enacting identical causative actions and differed only because the agent and patient roles were reversed. There were two independent variables: age as a between-participants variable with four levels (2-year-olds, 3-year-olds, 4-year-olds, and adults); and construction types as a within-participants variable with three levels (SVO construction, *ba*-construction, and subjectless *ba*-construction). The dependent variable was the number of times that each participant initially pointed at the correct animation in the test trials. In total, each participant completed an animal character identification stage, three screening trials, three real verb practice trials, and six test trials. A camcorder next to the participants and held by the experimenter recorded their pointing, and special care was taken not to record their faces.

Construction types were counterbalanced with each novel verb; therefore, three lists were derived. The three lists were SVO-*ba*-(s)*ba*, *ba*-(s)*ba*-SVO, and (s)*ba*-SVO-*ba*. The target side of the pointing was randomly determined across these three novel verbs within each list so that none of the lists had the same target sequences across these three types of transitive constructions. The participants were randomly assigned to one of three counterbalanced conditions.

Testing occurred in two labs at two national universities in Taipei, in a quiet room in the children’s nursery or in a quiet location in the children’s own homes. In all of the trials, the experimenters sat beside the participants to record their pointing, and the participants were told to concentrate on hearing the played sentences and movies on the computer screen. The order of the trials was as follows: animal character identification > novel verb screening trials > real verb practice trials > test trials.

The experimenters told the adult participants that they were going to investigate how they comprehended the heard utterances, and they told the child participants that they were going to play a game by pointing at the animations on the monitor. They were first shown an animation with eight animal characters (dog, cat, tiger, rabbit, bear, lion, hippo, and monkey), which were waving their hands and moving their legs. All of the participants correctly identified at least 75 percent of the animal characters.

Following the animal character identification, each participant completed three screening trials in a fixed order. The trial consisted of an animal performing a self-initiated action in one scene, but the animal was standing still in the other scene. The accompanying audio sentence was an intransitive sentence in Mandarin with a novel verb in its progressive aspect. For example, one trial consisted of a cat waving its hand in an S shape repeatedly in one scene. However, another cat was standing still in the other scene, and the participants heard the intransitive sentence *xiaomao zai kao-zhe* (“A little cat is kaoing”) and then *zhi-zhe xiaomao zai kao-zhe de difang* (“Point at the location where a little cat is kaoing”). All of the participants were encouraged to choose only one of the scenes—the one that they felt was the more appropriate of the two after they heard the sentence. If the participants (particularly the two-year-olds) hesitated to point at the scene, they were encouraged by the experimenters not to be afraid and received praise after they pointed. Otherwise, no other input was given to the participants. The participant’s first point was always used as the response. The arrangement of these trials was based on Noble et al.’s [30] suggestions that this trial exclude participants who could not pass a simple screening test that required them to point to a scene in which an animal is performing a self-initiated action. To pass this screening test, the participants needed to pass at least two of the three trials.

Developmental psycholinguists usually arrange familiarization trials before the test trials begin [7,21,30]. The real verbs in the SVO construction were used for the familiarization trials. The real verb practice trials consisted of three synchronized movies using three real verbs for the participants to choose from, i.e., *mou* “touch”, *wei* “feed”, and *ti* “kick”, while the synchronized accompanying scenes presented another action. The movies always involved an animal performing a familiar action for another animal in one scene, while the same animal performed a different action for the animal in the other scene. For example, one of the movies involved a bear pulling a hippo’s hand in one scene, while a bear was feeding the hippo an apple in the other scene. The accompanying audio sentences were *xiaoxiong weizhe hema* (“A little bear is feeding a hippo”) and *zhi-ze xiaoxiong weizhe hema de difang* (“Point at the location where a little bear is feeding a hippo”). Each participant completed three real verb practice trials in a fixed sequence.

After completing three real verb practice trials, each participant completed six novel verb test trials (one novel verb construction paired with two sets of scenes). These six novel verb trials consisted of three types of transitive constructions. Each movie involved an animal performing a novel action to the other animal in one scene, while the semantic roles of the two animals were reversed in another scene with the identical action. Two movies involving the same novel verb that denoted the same novel action with two different sets of animals were used for each type of transitive construction, as demonstrated above. The remaining two novel actions/novel verbs were paired with the remaining two types of transitive constructions: *ba*- and subjectless *ba*-constructions.

Although both real verb practice trials and test trials involved each of the two synchronized animations within a movie that had an animal (agent) performing a causal action to the other animal (the patient), these two trials differed in the following manners. First, all of the transitive constructions in the real verb practice trials used familiar verbs and the SVO construction only, whereas all of the movies in the test trials used novel verbs/actions; one novel verb was paired with the SVO construction, and the remaining two novel verbs were paired with transitive constructions other than the SVO construction. This arrangement decreases the possibility that the effects obtained in the test trials are derived from the preceding real verb practice trials and not from the participants’ general knowledge of syntax [22]. Second, different familiar actions were used in each of the synchronized animations in the real verb practice trials, whereas identical novel actions were used in each of the synchronized animation in the test trials. The child participants were praised for their pointing regardless of the accuracy rate that they attained (p90-p94, Hsu, 2017).

### Analysis

For every test trial, the recorded response was the participant’s first choice of matched scene after hearing the accompanying sentence. The data were coded by three trained coders; i.e., each of them coded one third of the data. Fifteen percent of each trained coders’ data were randomly selected and rechecked by one of the other two coders. The inter-rater reliability was 100%.

## Results and discussion

In this experiment, there were six trials for each child/adult. Table 2 demonstrates the number of participants who were correct in responding to a certain number of trials in the experiment for each age group. For example, the number to the right of the 2-year-olds and under trial 6 is 8, indicating that there were 8 2-year-olds who responded to 6 trials correctly or pointed to the side of the animation that matched their heard sentence in this experiment. A trend seemed to be able to be obtained: as age increased, more participants responded to all 6 of the trials correctly.

**Table 2:** The number of participants who responded correctly across ages.

Age	Number of Trials					
	6	5	4	3	2	1
2-year-olds	8	3	17	8	6	0
3-year-olds	12	16	7	3	4	1
4-year-olds	22	10	5	2	2	1
adults	37	4	1	0	0	0

Table 3 further demonstrates whether the participants responded to the trials correctly or not on the basis of the constructions that were examined in the experiment. Since each participant responded to two trials for each construction type, their responses were averaged in the Accuracy row, ranging from 1, indicating that they responded both trials correctly, to 0, indicating that they were correct in neither of the trials. The numbers below the row of (averaged) Accuracy are the number of participants whose averaged accuracies were performed in the corresponding cells. For example, the numbers 23 next to the 2-year-olds and 1 under the SVO construction indicate that there were 23 2-year-olds who pointed to both of the SVO trials correctly. The number 11 next to it indicates that 11 2-year-olds pointed to one of the two SVO trials correctly, and so on.

**Table 3:** The number of participants who responded correctly for each construction type across ages.

Age	Constructions								
	SVO			BA			(S)BA		
Accuracy	1	.5	0	1	.5	0	1	.5	0
2-year-olds	23	11	8	20	16	6	21	14	7
3-year-olds	28	8	7	29	8	6	29	10	4
4-year-olds	32	7	3	34	3	5	31	11	1
adults	41	1	0	40	2	0	39	3	0

**Fig 1:**
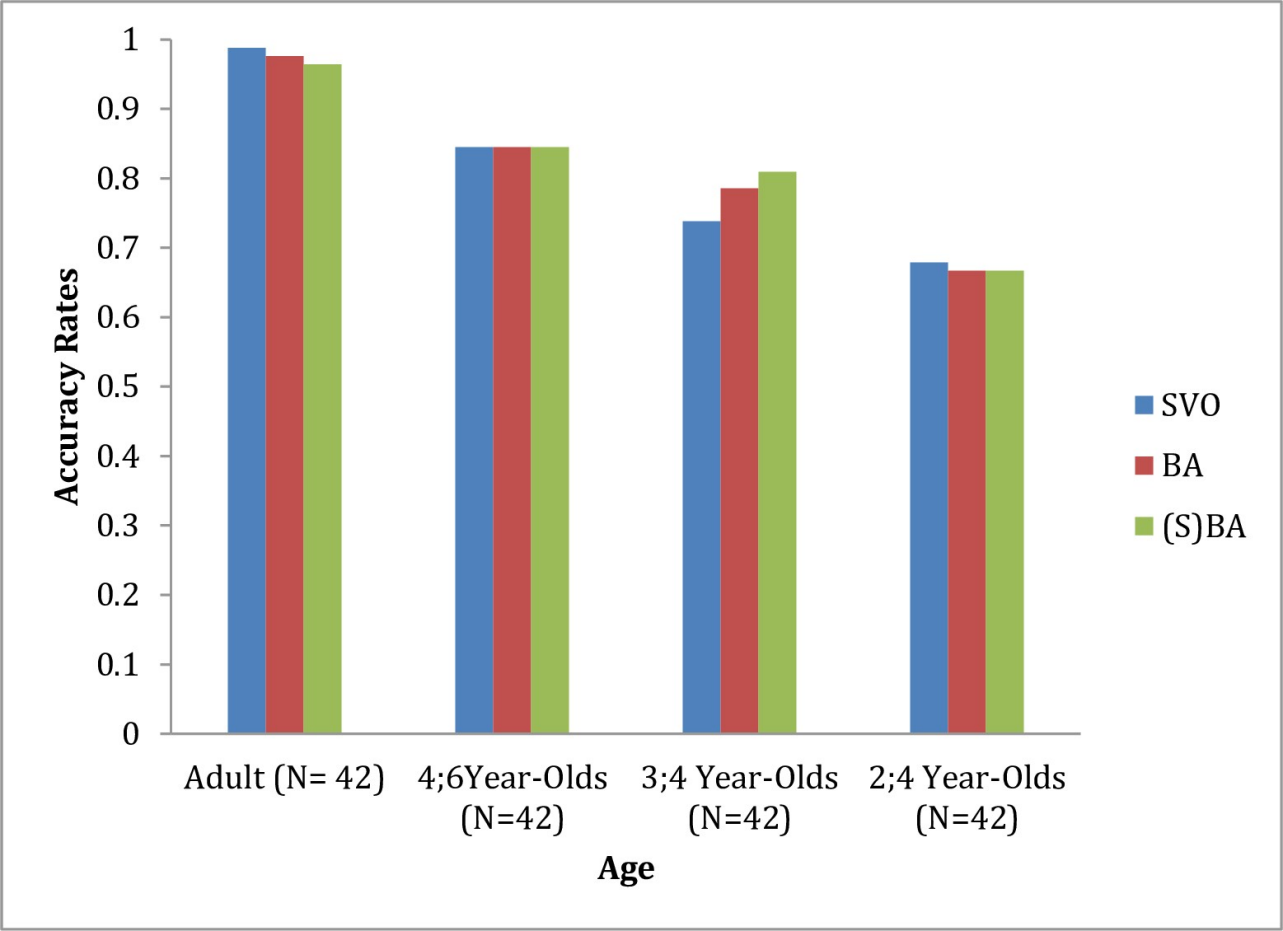
Mean proportion identifying the correct agent as a function of age and construction type.

Two-year-olds pointed to trials matched with the heard sentences approximately 68% of the time (SD = .40) for the SVO construction, 67% (SD = .36) for the *ba*-construction, and 67% (SD = .38) for the subjectless *ba*-construction. Three-year-olds pointed to trials matched with the heard sentences approximately 74% of the time (SD = .39) for the SVO construction, 79% (SD = .37) for the *ba*-construction, and 81% (SD = .33) for the subjectless *ba*-construction. Four-year-olds pointed to trials matched with the heard sentences approximately 85% of the time (SD = .30) for the SVO construction, 85% (SD = .34) for the *ba*-construction, and 85% (SD = .26) for the subjectless *ba*-construction. Adult participants pointed to trials matched with the heard sentences approximately 99% of the time (SD = .08) for the SVO construction, 98% (SD = .11) for the *ba*-construction, and 96% (SD = .13) for the subjectless *ba*-construction. Fig 1 shows the mean proportions of correct pointing to the correct agent as a function of age and construction type.

Data from the Mandarin-speaking participants of different ages were analyzed in two steps. In the first step, we analyzed the participants’ comprehension within a certain age group and investigated whether they employed syntactic representation of abstraction and whether the demonstration of syntactic abstraction differed across different transitive constructions. In the second step, we compared the participants’ performances across different age groups and transitive constructions.

To investigate whether Mandarin speakers demonstrated syntactic abstraction within a certain age group and whether they performed differently in different transitive constructions, two separate analyses were conducted for each examination. The first analysis compared the participants’ averaged accuracy rates of the two trials for each construction with the random chance level .5, and the second analysis compared the averaged accuracy rates for each construction to the others.

The results of the first analysis indicated that participants comprehended each construction beyond the chance level significantly. The effect of the 2-year-olds was (*T*(41) = 2.93, *p* = .006) with a 95% confidence interval (.05, .30) for the SVO construction, (*T*(41) = 3.00, *p* = .005) with a 95% confidence interval (.05, .28) for the *ba*-construction, and (*T*(41) = 2.86, *p* = .007) with a 95% confidence interval (.05, .28) for the subjectless *ba*-construction. The results of the second analysis, which used one-way ANOVA to compare the three construction types, indicated that there were no comprehension differences among them (*F*(2,123) = .014, *p* = .986). The effect of the 3-year-olds was (*T*(41) = 3.99, *p* < .001) with a 95% confidence interval (.12, .36) for the SVO construction, (*T*(41) = 4.59, *p* < .001) with a 95% confidence interval (.15, .38) for the *ba*-construction, and (*T*(41) = 5.55, *p* < .001) with a 95% confidence interval (.18, .39) for the subjectless *ba*-construction. The results of the second analysis, which used one-way ANOVA to compare the three construction types, indicated that there were no comprehension differences among them (*F*(2,123) = .18, *p* = .836). The effect of the 4-year-olds was (*T*(41) = 7.40, *p* < .001) with a 95% confidence interval (.25, .44) for the SVO construction, (*T*(82) = 6.58, *p* < .001) with a 95% confidence interval (.24, .45) for the *ba*-construction, and (*T*(82) = 8.65, *p* < .001) with a 95% confidence interval (.26, .43) for the subjectless *ba*-construction. The results of the second analysis, which used one-way ANOVA to compare the three construction types, indicated that there were no comprehension differences among them (*F*(2,123) = .000, *p* = 1). The effect of adults was (*T*(41) = 41.00, *p* < .001) with a 95% confidence interval (.46, .51) for the SVO construction, (*T*(41) = 28.64, *p* < .001) with a 95% confidence interval (.44, .51) for the *ba*-construction, and (*T*(41) = 23.09, *p* < .001) with a 95% confidence interval (.42, .50) for the subjectless *ba*-construction. The results of the second analysis, which used one-way ANOVA to compare the three construction types, indicated that there were no comprehension differences among them (*F*(2,123) = .517, *p* = .598).

Fig 1 indicates that Mandarin-speaking 2- to 4-year-olds and adults exhibited a similar pattern in comprehending the SVO construction, the *ba*-construction, and the subjectless *ba*-construction. The statistical analyses generally indicated that Mandarin speakers, regardless of their ages, comprehend these three types of construction at a rate better than chance; the results did not differ based on the type of construction. To investigate whether there are any developmental changes among ages, two-way mixed ANOVA was conducted. The between-participants variable was age (2 vs. 3 vs. 4 vs. adults), the within-participants variable was construction type against chance (SVO vs. BA vs. (S)BA vs. chance), and the dependent variable was the average correct proportion identifying the matching video clips. Fig 1 suggests that Mandarin speakers performed differently at different ages, as supported by the main effects of age (*F*(3,164) = 19.06, *p* < .001, *η*^*2*^ = .26) and construction type (*F*(1,164) = 146.86, *p* < .001, *η*^*2*^ = .73) and by the interactive effects between age and construction type (*F*(3,164) = 5.28, *p* < .001, *η*^*2*^ = .088). The interactive effect occurs because Mandarin-speaking preschoolers performed differently from their adult counterparts (two- and three-year-olds vs. adults, both *p* < .001; four-year-olds vs. adults, *p* = .013), and two-year-olds performed differently from their four-year counterparts (*p* < .001), whereas two-year-olds and three-year-olds and three-years-olds and four-year-olds did not differ from one another (both *p* > .05).

However, a general picture concerning preschoolers’ demonstration of syntactic abstraction emerged. Mandarin-speaking preschoolers aged from 2 to 4 years old comprehended the SVO construction, *ba*-construction, and subjectless *ba*-construction at rates significantly better than chance, suggesting that they are equipped with a verb-general abstract representation of syntax to interpret incoming strings of utterances. They also employed a pattern of comprehension of these transitive constructions similar to adults’ competence in comprehending transitive constructions; i.e., preschoolers and adults comprehended the constructions equally well using the first-NP as agent and patient-marked case *ba*.

The findings support the structure-mapping theory of young children’s language acquisition in the following manner. First, Mandarin-speaking children, at least when they are aged 2–4 years old, employed a partial but abstract representation of syntax to interpret the incoming strings of utterances; this result may occur independently of input characteristics, which is evidence against the graded representation theory.

Second, comparing the results with Chan et al.’s [16] findings regarding SVO construction with novel verbs, we found that the methodology used to examine young children’s exhibition of syntactic abstraction plays a critical role. The reason that Cantonese-speaking 2-year-olds in Chan et al.’s [16] study did not demonstrate syntactic abstraction with novel verbs in the SVO construction and neutralized animacy of the NPs is most likely because the act-out methodology was overwhelming to these young children. Using a less demanding methodology, such as FCPP, allows children to demonstrate their linguistic abstraction better.

Third, comparisons between children’s performance in Chan et al. [16] and adults’ performance in Liu et al. [37] and Li et al. [3] suggest that Mandarin-speaking young children do not always use a *weaker* representation than adults do, and they occasionally use a *stronger* one. Cantonese-speaking children aged 2;6–4;6 years old in Chan et al.[16] performed much better in the cue-in-conflict construction (the IVA construction, e.g., “The present tams the chicken”), with 53–78% correctly identifying the first NP as agent, more than did the adults (10–35%) in Liu et al. [37] and in Li et al. [3]. Cantonese/Mandarin-speaking children tend to use word order knowledge that treats the first NP as the agent and the second NP as the patient, as the structure-mapping theory has claimed [21].

The findings concerning the *ba*-construction and subjectless *ba*-construction also contrasted with what Li et al. [3] found. Mandarin speakers are good at taking advantage of *ba* as a case marker to comprehend the (S)baOV construction even when it has low cue validity. Nevertheless, Chan et al.’s results and our findings differ from those of adult studies. The difference might exist because those studies used familiar verbs [3,37], and the adults’ performance was confined by their previous experience with particular verbs.

## Experiment 2

The passive construction constitutes a major problem throughout English-speaking young children’s early acquisition of syntax because of its low frequency, its lack of reliable cues to denote the construction [38] and its mapping order of the thematic role against English’s predominant agent-patient mapping between the thematic role and grammatical functions. The difficulties that English-speaking children encounter have been discussed in more detail by Huang et al. [26] and Messenger, Branigan, McLean, and Sorace [39]. As a result, both the structure mapping account and the graded representations account predict the less optimal performance that English-speaking young children might demonstrate.

The Mandarin passive construction has been reported to be less common than the English passive construction [26]; the occurrence of Mandarin passive construction is one-tenth that of the English passive construction. However, the Mandarin passive construction has a reliable case marker cue, the *bei* marker, which facilitates Mandarin-speaking young children’s acquisition of it. Thus, the graded representation account, which was based on the Competition Model [13], would have difficulty in predicting Mandarin-speaking young children’s performance with the construction because it is far less common. However, it still has a reliable cue for comprehension, i.e., case marking that plays a significant role in young children’s acquisition of syntax [28]. Conversely, the structure mapping account would predict that young children would perform worse in the *bei*-construction than in the *ba*-construction because the latter conforms to the agent-patient role assignment, whereas the former does not. Indeed, such a prediction was borne out in Huang et al. [26]. They tracked Mandarin-speaking 5-year-olds’ eye movements when they were comprehending the Mandarin full *ba*- and *bei*-construction when both NPs were animate and recorded their act-out performances during the comprehension of these sentences. The results indicated that Mandarin-speaking five-year-olds performed better on the *ba*-construction than on the *bei*-construction in both tasks. In the structure mapping account, Mandarin-speaking young children revised their looks for the *bei*-construction more when the first NP was a full NP than when it was a pronoun NP. Although these children demonstrated adult-like proficiency in the eye-tracking task with both constructions, their performance with the *bei*-construction, but not with the *ba*-construction, suffered in the act-out measure. This difference appeared to strengthen the effects of tasks on young children’s demonstration of the abstraction of syntax, as mentioned earlier.

Nonetheless, Zeng, Mao, and Duan [40] recently reported that Mandarin-speaking children performed equally well with the active, short passive and long passive constructions when both NPs are animate. The 4-year-olds were approximately 75–80% accurate, and the five-year-olds were approximately 95% accurate in a pointing task in which a series of four pictures were presented to the children; the children were asked to point at the picture that matched the incoming sentence. The participants’ performance with the passive constructions in Zeng et al. [40] was better, at least numerically, than the 5-year-olds and even adults in Huang et al.’s [26] study. Furthermore, Li et al. [3] suggested that Mandarin-speaking adults comprehended *bei*-construction better than *ba*-construction because the *ba* marker in *ba*-construction has a homophonous particle, *ba*, which interferes with Mandarin adults’ comprehension.

All of the discrepancies discussed above could have resulted from the various task demands exerted on Mandarin-speaking adults and young children, from the different designs of the tasks, such as the blocking design versus non-blocking design [26], and from the constructions that were interpreted in terms of different verb types. To investigate the *ba*-, short-*bei*, and long-*bei*-constructions, to avoid the interference of the verb type and to choose an appropriate task for both children and adults, this study investigates how Mandarin-speaking 2- to 4-year-olds and adults comprehended the SVO-, *ba*-, short passive, and long-passive constructions with novel verbs when both NPs are animate in a forced-choice pointing paradigm.

## Methods

### Participants

Twenty-eight 2-year-olds (range = 1;11–2;11, mean age = 29.75 months, SD = 3.01, 13 boys), 28 3-year-olds (range = 3;0–3;11, mean age = 42.79 months, SD = 3.19, 14 boys), 28 4-year-olds (range = 4;0–4;11, mean age = 53.29 months, SD = 3.68, 15 boys), and 28 adults (range = 19–35, mean age = 22.86, SD = 3,83, 12 men) participated in a language comprehension task using FCPP. All of the participants were speakers who were dominant in Mandarin Chinese, with less than 20% exposure to a language other than Chinese dialects, and they had no language difficulties. The procedures for the recruitment of the participants, the locations of the experiments, and the criteria for participant selection for the final analysis were identical to those in Study 1. Nine 2-year-olds, 5 3-year-olds, 2 4-year-olds, and 2 adults were excluded because they did not meet the criteria above. Another 4 2-year-olds were excluded due to fussiness.

This study involving human participants has been approved by the author’s Institutional Review Board (IRB) in Taiwan (Research Ethics Committee, National Taiwan University). The approval numbers are NTU-REC No.: 201312ES034 and NTU-REC No.: 201506ES028. Informed written consent has been obtained from the participants for the adult participants and from the child participants’ parents.

### Materials, design, counterbalancing, and procedures

In addition to the three novel verbs used in the previous study, one more novel verb, *pya*, was added to this study to accommodate the fourth construction in the current study. The novel verb *pya* also depicted a prototypical causal event with a clear end point. In this event, an animal placed another animal (from the animal pool in the previous study) on the flat surface of a stool-like apparatus, raised this apparatus, and then lowered it to the floor. Two trials of this verb were created in Flash Player and exported as movies to Dreamweaver. The movies consisted of two synchronized side-by-side animations and were presented twice on a laptop. The events in these two synchronized animations were identical except when the animal characters were flipped. Four lists were created to counterbalance the design in which each novel verb could be paired in each of the four lists with one of the four constructions: the SVO construction, the *ba*-construction, the long-passive construction, and the short passive construction.

### Analysis

The coding criteria were identical to those in the previous study, and the inter-rater reliability was again 100%.

## Results and discussion

There were eight trials for each child/adult in this experiment. Table 4 shows the numbers of participants that were correct in responding a certain number of trials for each age group. For example, the number to the right of the 2-year-olds and under trial 8 is 5, indicating that there were 5 2-year-olds who responded to 8 trials correctly or pointed to the side of the animation that matched their heard sentence in this experiment. A trend seemed to be able to be obtained, though this outcome might not be the case for 2- and 3-year-olds in the experiment: as the age increased, more participants responded to all of the 8 trials correctly.

**Table 4:** The number of participants who responded correctly across ages.

Age	Number of Trials							
	8	7	6	5	4	3	2	1
2-year-olds	5	5	7	1	6	2	2	0
3-year-olds	8	4	3	5	5	2	1	0
4-year-olds	12	6	6	3	1	0	0	0
adults	23	4	1	0	0	0	0	0

Table 5 further indicates whether or not the participants responded to the trials that were matched to the sentences they had heard. Since each participant responded to two trials for each construction type, their responses were likewise averaged in the Accuracy row, ranging from 1 when they responded to both trials correctly to 0 when they were correct in neither of the trials. The numbers below the row of (averaged) Accuracy are the numbers of participants whose averaged accuracies are presented in the corresponding cells. For example, the number, 17 next to the 2-year-olds and 1 under the SVO construction shows that there were 17 2-year-olds who pointed to both of the SVO trials correctly. The number 4 next to it shows that 4 2-year-olds pointed to one of the two SVO trials correctly, and so on.

**Table 5:** The number of participants who responded correctly for each construction type across ages.

Age	Construction Type											
	SVO			BA			Long BEI			Short BEI		
Accuracy	1	.5	0	1	.5	0	1	.5	0	1	.5	0
2-year-olds	17	4	7	18	2	8	17	4	7	17	8	3
3-year-olds	19	6	3	19	2	7	16	8	4	16	7	5
4-year-olds	19	6	3	25	0	3	22	3	3	24	4	0
adults	27	1	0	27	1	0	25	3	0	27	1	0

**Fig 2:**
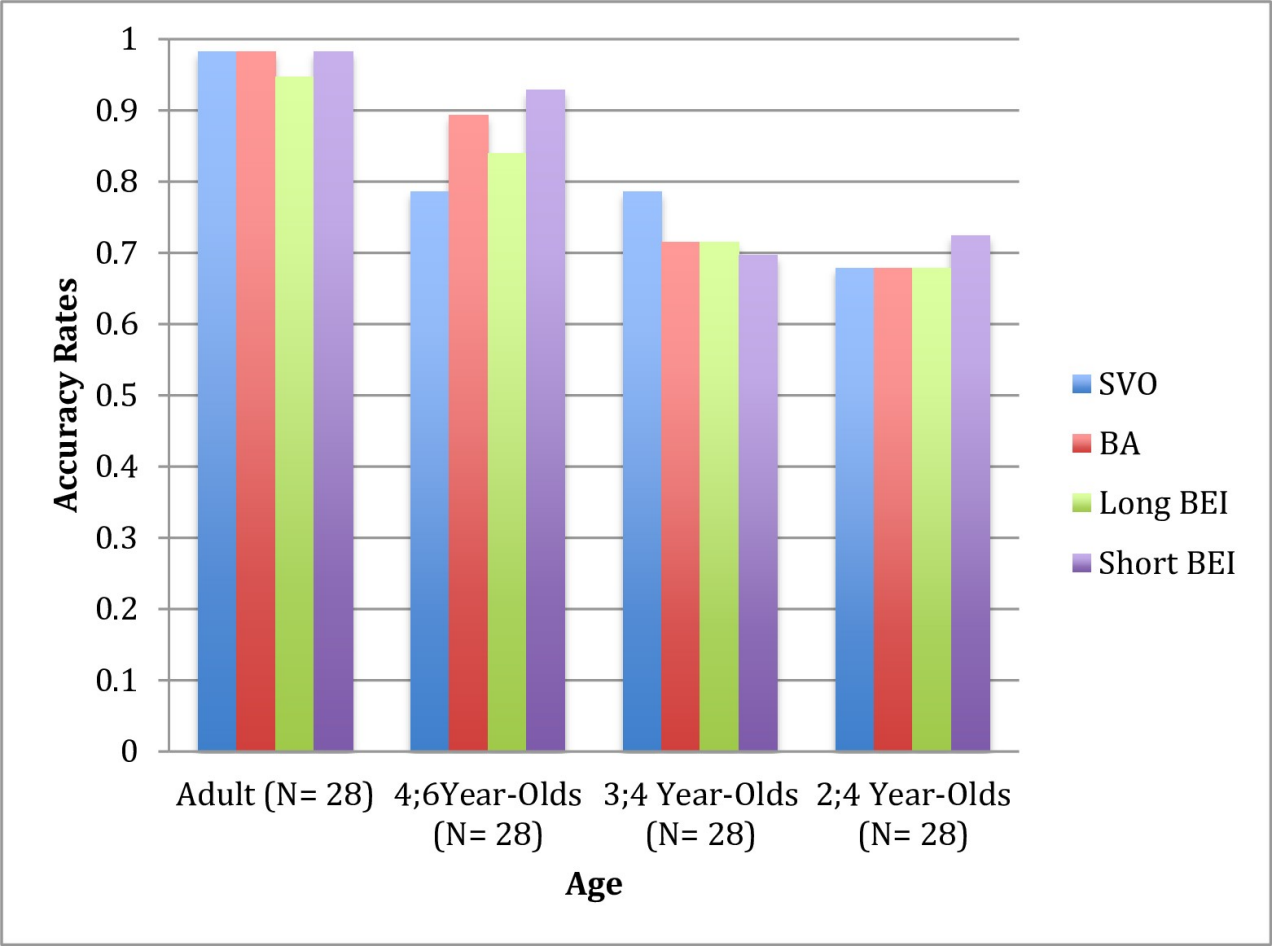
Mean proportions of identifying the correct agent as a function of age and construction type.

The two-year-olds pointed to trials matched with the heard sentences approximately 68% of the time (SD = .43) for the SVO construction, 68% (SD = .46) for the *ba*-construction, 68% (SD = .43) for the long *bei*-construction, and 72% (SD = .35) for the short *bei*-construction. Three-year-olds pointed to trials matched with the heard sentences approximately 79% of the time (SD = .35) for the SVO construction, 71% (SD = .44) for the *ba*-construction, 71% (SD = .37) for the long *bei*-construction, and 70% (SD = .39) for the short *bei*-construction. Four-year-olds pointed to trials matched with the heard sentences approximately 79% of the time (SD = .35) for the SVO construction, 89% (SD = .31) for the *ba*-construction, 84% (SD = .33) for the long *bei*-construction, and 93% (SD = .18) for the short *bei*-construction. Adult participants pointed to trials matched with the heard sentences approximately 98% of the time (SD = .09) for the SVO construction, 98% (SD = .09) for the *ba*-construction, 95% (SD = .16) for the long *bei*-construction, and 98% (SD = .09) for the short *bei*-construction. Fig 2 shows the mean proportions of correct pointing to the correct agent as a function of age and construction type.

We followed the steps applied in the previous study to analyze the data in this study. We initially analyzed the participants’ comprehension to determine whether they comprehended these constructions better than at the chance level and whether their demonstrations differed across different transitive constructions. Then, we compared the participants’ performance across the different age groups and transitive constructions. Fig 2 shows the mean proportions of correct pointing to the correct agent as a function of age and construction type.

To understand whether these Mandarin speakers comprehended the constructions better than at the chance level within a certain age group and whether they performed differently in these transitive constructions, four t-tests were conducted. Because each participant had two opportunities to respond to one construction type, the accuracy rates of these two trials were averaged. Then, the proportions of accuracy of each construction type (SVO vs. *ba*-construction vs. long passive construction vs. short passive construction vs. chance) were compared with the random chance level .5. To investigate whether children and adults performed differently with respect to these four constructions, mixed two-way ANOVA was applied, with the age as the between-participants independent variable (2 vs. 3 vs. 4 vs. adults), the construction as the within-participants independent variable (SVO vs. *ba* vs. long *bei* vs. short *bei*), and the proportions of accuracy of each construction compared to the chance level as the dependent variable.

Fig 2 indicates that Mandarin-speaking 2- to 4-year-olds and adults exhibited a similar pattern in comprehending the SVO construction, the *ba*-construction, and the long passive construction, although they appeared to comprehend the short passive construction better at the ages of 2 and 4 years old, i.e., numerically more than 4 percent, than they did the other constructions. The statistical analyses indicated that Mandarin speakers, regardless of their age, comprehend these four types of construction at a rate better than would be predicted by random chance. The statistical results indicated that the 2-year-olds comprehended these four constructions better than would be predicted by random chance, i.e., *T*(27) = 2.17, *p* = .039 with a 95% confidence interval (.001, .39) for the SVO construction; *T*(27) = 2.07, *p* = .047 with a 95% confidence interval (.002, .320) for the *ba*-construction; *T*(27) = 2.17, *p* = .039 with a 95% confidence interval (.011, .350) for the long passive construction; and t(27) = 3.81, *p* = .001 with a 95% confidence interval (.12, .38) for the short passive construction. The results of the second analysis, which used one-way ANOVA to compare the four construction types, indicated that there were no comprehension differences among them (*F*(3,108) = .20, *p* = .90). The results indicated that the 3-year-olds comprehended the four constructions better than would be predicted by random chance, i.e., *T*(27) = 4.38, *p* < .001 with a 95% confidence interval (.15, .42) for the SVO construction; *T*(27) = 2.58, *p* = .016 with a 95% confidence interval (.04, .38) for the *ba*-construction; *T*(27) = 3.06, *p* = .005 with a 95% confidence interval (.07, .36) for the long passive construction; and *T*(27) = 2.65, *p* = .013 with a 95% confidence interval (.04, .35) for the short passive construction. The results of the second analysis, which used one-way ANOVA to compare the four construction types, indicated that there were no comprehension differences among them (*F*(3,108) = .29, *p* = .83). The 4-year-olds and adults also comprehended these constructions better than would be predicted by random chance, i.e., for the 4-year-olds, *T*(27) = 4.38, p < .001 with a 95% confidence interval (.15, .42) for the SVO construction; *T*(27) = 6.60, p < .001 with a the 95% confidence interval (.27, .52) for the *ba*-construction; *T*(27) = 5.36, p < .001 with a 95% confidence interval (.21, .47) for the long passive construction; and *T*(27) = 12.73, p < .001 with a 95% confidence interval (.36, .50) for the short passive construction. The results of the second analysis, which used one-way ANOVA to compare the four construction types, indicated that there were no comprehension differences among them (*F*(3,108) = 1.21, p = .31). For the adults, the results were T(27) = 27, *p* < .001 with a 95% confidence interval (.45, .52) for the SVO construction; *T*(27) = 27, *p* < .001 with a 95% confidence interval (.45, .52) for the *ba*-construction; *T*(27) = 15, *p* < .001 with a 95% confidence interval (.37, .51) for the long passive construction; and *T*(27) = 27, *p* < .001 with a 95% confidence interval (.45, .52) for the short passive construction. The results of the second analysis, which used one-way ANOVA to compare the four construction types, indicated that there were no comprehension differences among them (*F*(3,108) = .69, *p* = .56).

To investigate whether there were any developmental changes among ages, two-way mixed ANOVA was conducted. Fig 2 suggests that, although Mandarin speakers comprehended these four constructions better than at the random chance level (*F*(1,108) = 80.96, *p* < .001, *η*^*2*^ = .43), they performed differently at different ages, as supported by the main effects of age (*F*(3,108) = 13.46, *p* < .001, *η*^*2*^ = .27) and a significant effect of the interaction between the constructions and age (*F*(3, 108) = 4.51, *p* = .0 05, *η*^*2*^ = .11). Bonferroni’s post hoc analysis indicated that the comparison between Mandarin-speaking 2-year-olds and 3-year-olds, and the comparison between four-year-olds and adults did not differ from each other in comprehending these four types of constructions (both *p* > .05), whereas two-year-olds’ and three-year-olds’ performance was different from the 4-year-olds’ and adults’ (all *p* < .05).

Both the structure mapping and the graded representation accounts partially predicted the results obtained in this study, although the structure mapping account predicted that Mandarin-speaking young children could form abstraction of syntax using the partial representation formed by the nouns and the tendency to treat the first NP as agent. However, even when the first NPs in the long and short passive constructions are interpreted as patient, the tendency did not interrupt the children’s comprehension; in contrast, they comprehended such constructions at least as well as the constructions such as the SVO construction and the *ba*-construction, which confirm the first NP as agent tendency. The reliability of the *bei* marker that denotes the patient and that can additionally denote the agent allows Mandarin speakers to comprehend the Mandarin passive constructions rather well. Its 100% cue reliability determines the interpretation of the passive construction at a level similar to the other two constructions.

The results are in line with Zeng et al.’s [40] results but counter to Huang et al.’s [26], in which Mandarin speakers comprehended *ba-*constructions better than they did *bei*-constructions. The discrepancies between these studies and Li et al.’s study, in which the participants conversely comprehended the *bei*-construction better than they did the *ba*-construction, can be attributed to differences in the experimental designs and methodologies employed among these studies. Huang et al. [26] employed act-out measures to examine Mandarin-speaking adults’ and 5-year-olds’ comprehension, and the results indicated that even adults did not perform very well. In contrast, Zeng et al. [40] and the current study used a task that primarily used pointing as a measure, which led to much higher rates of accuracy on the *bei*-constructions. Moreover, *bei*-construction might have been overestimated in Li et al.’s [3] studies; as Huang et al. [26] noted, Li et al. [3] employed a blocking design that might have boosted the participants’ performance of the *bei*-constructions. In addition, compared to the results from Zeng et al. [40], the current study and Li et al. [3] suggest that the five-year-olds’ comprehension or ‘finding the agent’ performance in the passive construction can occasionally be better than that of adults, which again argues that children’s representations are not always weaker than those of adults. For example, five-year-olds in the study by Zeng et al. [40] who comprehended these constructions were accurate more than 90% of the time compared to an accuracy rate of approximately 80% in adults in Li et al.’s study [3]. Consequently, this comparison suggests that Abbot-Smith et al. [13] deemed it inappropriate to use the graded representations of weak vs. strong representations [41] to pinpoint the difference between Mandarin-speaking young children’s and adults’ exhibition of the syntactic representation of abstraction because children could perform better than adults when the same type of syntactic construction was examined.

The results suggest that regardless of age, Mandarin speakers can comprehend the SVO construction, the *ba*-construction, and the two types of passive constructions equally well. Moreover, the fluctuations in the performance of comprehension in these constructions could be attributed to the experimental design and methodologies that were used to investigate the processing.

## General discussion and conclusions

The current study attempted to investigate when Mandarin-speaking young children demonstrate syntactic abstraction and whether this abstraction is affected by input characteristics, such as the number of cues, cue availability, cue reliability, and cue validity, using five types of Mandarin transitive constructions: the *ba*-construction, the SVO construction, the subjectless *ba*-construction, the long passive construction, and the short passive construction.

The results of this study emphasize the importance of the methodology employed when investigating young children’s demonstration of abstraction of syntax. Fisher [6] argued that young children’s demonstration of syntactic competence can be obscured by demanding methodologies [16,22,29]. Their claim has indeed gained support from direct comparison between act-out and FCPP in Dittmar et al. [22], comparison of the eye-tracking technique and act-out measure in Huang et al. [26] and indirect comparisons between this study and Chan et al. [16] and among Huang et al.’s, Zeng et al. [40] and the current study. The findings obtained in the current study contradict those of Chan et al. [16], in which Cantonese (Chinese)-speaking children aged between 2;6 and 3;6 year sold were unable to use word order cues only (when animacy cues were neutralized) to identify the agent in the SVO construction with novel verbs in an act-out paradigm. The findings also contrast with Huang et al.’s [26] study of Mandarin-speaking 5-year-olds’ performance of passive construction in the act-out measure, but they are in line with Zeng et al.’s [40] Mandarin-speaking 4- and 5-year-olds’ and adults’ performance with short and long passive construction in a pointing task similar to that used in this study. The difference in the demonstration of verb-general syntactic knowledge in young children aged between 2;6 and 3;6 years old for the SVO construction and 4- and 5-year-olds for the passive construction can be attributed to the methodological differences between previous studies: Chan et al. [16], Huang et al. [26], and Zeng et al. [40].

In light of syntactic bootstrapping studies and conjoined agent intransitive studies [9,11,12,21,30,42], one might doubt whether Mandarin-speaking two-year-olds are indeed able to employ a representation of abstract syntax to understand the agent-patient relationship. One could argue that Mandarin-speaking two-year-olds can use syntactic bootstrapping to assign distinct roles to the two NPs embedded in the *ba*-construction, plus use their bias to treat first-named NPs as agents to comprehend the *ba*-construction, exactly like they misinterpreted the conjoined agent intransitive constructions as transitive constructions. Because the Mandarin *ba*-construction and the English conjoined agent intransitive have similar structures (i.e., NP and/*ba* NP VP), Mandarin-speaking children’s misinterpretation leads to their accurate comprehension of the *ba*-construction. Nonetheless, the results obtained in Study 2 argue against such an alternative explanation and assert that Mandarin-speaking young children indeed possess a representation of the syntax of the case marker *ba*. First, the results concerning the conjoined agent intransitive in English-speaking 21- and 25-month-olds reflected a transitional phase for these toddlers’ construction of the correct representations [21]. In other words, the misinterpretation of the *ba*-construction-like interpretation is not an accurate representation because children 25 months of age and older can interpret this construction differently and accurately. In contrast, Mandarin speakers should employ this interpretation to comprehend the *ba*-construction, and this study shows that Mandarin-speaking 28-month-olds possess this abstraction. Second, the interpretation of the subjectless *ba*-construction in Mandarin-speaking 28-month-olds further lends support to their ability to employ the case marker to comprehend sentences. There is no explicit agent in this construction, but Mandarin children can rely on the *ba*-marked patient to indicate the agent implicitly. That they comprehend both this method and the *ba*-construction with the explicit subject indicates that they have a firm representation of abstraction with the *ba*-construction. The results suggest that Mandarin-speaking young children can integrate language-specific cues, in addition to syntactic bootstrapping [8], to guide them in the their subsequent learning of verbs, particularly when the verbs are impoverished/novel (cf. [43]).

In contrast, it is likely that these preschoolers’ performance is primarily based on structural priming of the SVO constructions, which have been used in practice trials since Hsu [32,44], who found that Mandarin-speaking preschoolers can be primed with the SVO construction and *ba*-construction. Although preschoolers’ comprehension in Experiment 1 could have been involved with structural priming, it is untenable to argue that their abstraction of these constructions was due to structural priming for the following two reasons. First, structural priming has been argued and demonstrated to be a side effect of children’s formation of abstract syntactic representations. As a result, structural priming occurs only when these children are equipped with abstract representations. Second, the results in Experiment 2, in which the preschoolers comprehended the *ba*-construction, as well as the *bei*-constructions, suggested that their abstraction of comprehension of these constructions was due to their employment of structural cues, such as word order, *ba*- and *bei*-markers and their systematic correspondence with the thematic roles of agent and patient. Whether this identification can lead to further structural priming among SVO, *ba*- and the two types of *bei*-constructions in Mandarin Chinese among preschoolers requires further research to determine.

Although the results of the current study support the view that sentence comprehension can occur independent of the frequencies of the constructions [45,46], the consensus that young children’s performance is not as good as that of adults, either defined as graded or as partial/incomplete representation, calls for an explanation. Two accounts are juxtaposed. One is a structural frequency-based explanation, particularly for young preschoolers. Although the cue availabilities, reliabilities, and validities cannot fully capture these young children’s comprehension patterns, it is worth mentioning that the role of frequency cannot be downplayed. Early discussion suggested that toddlers at the age of 19 months old can employ syntactic bootstrapping to take advantage of correspondences between word order and thematic roles to interpret the relationships of agents and patients. Nonetheless, these toddlers might be unaware of the structural functions of *ba* and *bei* because they are quite infrequent in their input. As they are exposed to input by adults or caregivers around them, they simultaneously note that word order is not a very reliable cue to identify the agent and patient relationship because of the massive argument ellipsis in Mandarin, whereas the structural markers *ba* and *bei* are very reliable. As a result, they can develop a strategy in light of the increasing exposure to input; i.e., they use word order when no case marking cues are available, but they prioritize case cues when they are available. In this scenario, input influences and facilitates young children’s increasing comprehension accuracy and explains the developmental trajectories of young preschoolers and adults.

The other account is a long-held maturational theory that can be used to explain the gradation of performance among Mandarin speakers across different ages. Sakai’s [47] report of the maturation rate of the brain regarding language appears to mesh well with the data presented in this study. Differences across different ages among the 2- to 4-year-olds and adults can be attributed to a quantitative difference because they used the same grammatical representation for comprehension, rather than a qualitative change from no representation to some abstract representation, as the graded representation accounts claimed. In addition, the rate of difference was likely due to the maturational constraint, rather than the input characteristics (see also Johnson & Newport [48,49]).

The results present strong evidence that Mandarin-speaking children and adults use their sensitivity to the objective-case marker *ba* and the agentive case marker *bei* to guide sentence interpretation in a language that enables massive argument ellipsis. Although these representations might be error-prone due the influence of input, maturation or both, a similar pattern between children and adults in comprehending these transitive constructions suggests that young children employ structure-sensitive representation by the age of 2 years old.

## Supporting information

S1 Experiment (XLSX)

S2 Experiment (XLSX)
